# Delineating sex-specific circulating host response signatures associated with COVID-19 severity and mortality

**DOI:** 10.1016/j.isci.2024.111150

**Published:** 2024-10-11

**Authors:** Nick Keur, Antine W. Flikweert, Isis Ricaño-Ponce, Anneke C. Muller Kobold, Simone van der Sar-van der Brugge, Izabela A. Rodenhuis-Zybert, Kieu T.T. Le, Matijs van Meurs, Marco J. Grootenboers, Peter H.J. van der Voort, Peter Heeringa, Vinod Kumar, Jill Moser

**Affiliations:** 1 Department of Internal Medicine and Radboud Center for Infectious Diseases, Radboud UMC, Nijmegen, the Netherlands; 2 Department of Pulmonary Medicine, Amphia Hospital, Breda, the Netherlands; 3 Department of Critical Care, University of Groningen, University Medical Center Groningen, Groningen, the Netherlands; 4 Department of Laboratory Medicine, University of Groningen, University Medical Center Groningen, Groningen, the Netherlands; 5 Department of Pathology & Medical Biology, University of Groningen, University Medical Center Groningen, Groningen, the Netherlands; 6 Department of Medical Microbiology & Infection Prevention, University of Groningen, University Medical Center Groningen, Groningen, the Netherlands; 7 Department of Genetics, University of Groningen, University Medical Center Groningen, Groningen, the Netherlands

**Keywords:** Molecular biology, Microbiology, Virology

## Abstract

Male SARS-CoV-2-infected patients have higher hospitalization rates, ICU admissions, and mortality compared to females, yet with unclear underlying mechanisms. We investigated the influence of biological sex on COVID-19 severity and patient outcomes. We profiled 41 circulating host response markers and identified differentially regulated proteins based on disease severity using covariates, such as sex, age, BMI, diabetes, and corticosteroid administration. IL-8, D-dimer, S100B, IL-6, Angpt-2, MMP-8, TNF-R1, u-PAR, u-PA, osteopontin, IL-13, TNF-α, pentraxin-3, P-selectin, fractalkine, and SP-D levels were elevated in critically ill COVID-19 males compared to severe cases. In contrast, IL-8, D-dimer, IL-6, Angpt-2, Tie-2, uPAR, and SP-D were higher in females with critical-COVID-19 than in severe cases. Notably, D-dimer, IL-6, pentraxin-3, and S100B were associated with male mortality, yet none of the measured plasma proteins associated with female mortality. Our study delineated distinct sex-specific plasma protein signatures linked to the severity and mortality of COVID-19 patients.

## Highlights

• Circulating host response proteins correlated with age, BMI, and sex in COVID-19 patients
• Critical COVID-19 patients showed shared and sex-specific host response signatures
• D-dimer, IL-6, pentraxin-3, and S100B are male-specific mortality markers in COVID-19

## Graphical abstract

**Figure:**
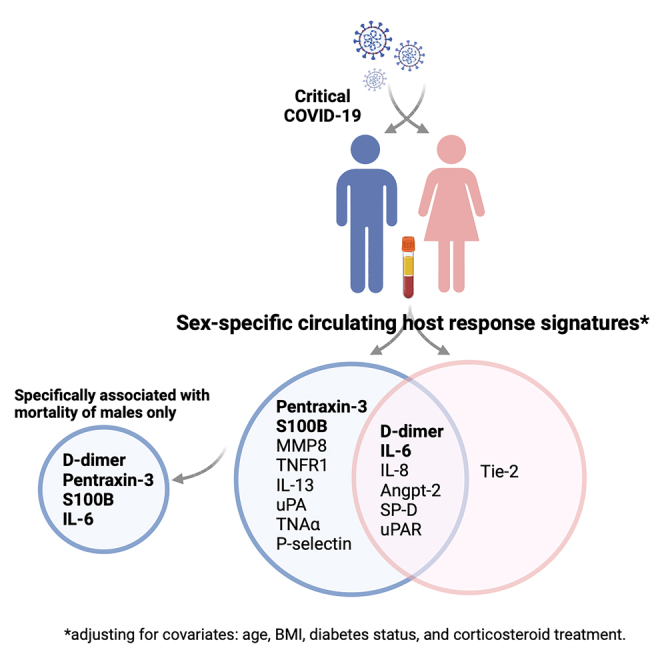

## Introduction

SARS-CoV-2 infection in humans can lead to various manifestations ranging from mild symptoms to severe and lethal disease.^1^ Patients with severe coronavirus disease 2019 (COVID-19) develop acute respiratory distress syndrome (ARDS), requiring invasive mechanical ventilation and other organ support in the intensive care unit (ICU). Although men and women have similar infection rates,^2^ recent large-scale epidemiological studies have identified age and sex as risk factors for the development of severe COVID-19.^3^^,^^4^ Biological sex is known to influence the risk and susceptibility to critical illness in COVID-19^5^ and is becoming increasingly apparent owing to intense research efforts to understand the pathophysiology of this disease. SARS-CoV-2-infected males are more likely to require hospitalization and ICU care,^3^^,^^4^ whereas females have worse functional outcomes after discharge and are more likely to develop long-COVID syndrome.^6^ Globally, males develop more severe disease and display increased mortality compared to females.^7^ The mechanisms underlying the development of more severe COVID-19 in males and the differentiating effects of sex on the clinical trajectory of hospitalized SARS-CoV-2 infected patients are currently poorly understood. Males tend to have more comorbidities,^8^ which might account for these differences; however, sex differences in COVID-19 severity persist even after controlling for comorbidities.^9^ Understanding and identifying sex-based differences and the mechanisms involved are crucial to help predict patient outcomes and facilitate clinical decision-making in terms of treatment and precision therapies for hospitalized COVID-19 patients.

Several cross-sectional proteomic studies have been conducted to identify biomarkers to predict COVID-19 severity.^10^^,^^11^^,^^12^^,^^13^^,^^14^^,^^15^^,^^16^ These studies identified proteins involved in multiple pathways, including inflammation (IL-6, TNF, IL-8, and CXCL6), immune cell activation (CD244 and CD40), coagulation (D-dimer, fibrinogen, and factor-D), apoptosis (TRAIL and SCF), and endothelial dysfunction (ACE2 and KRT-19). Sex differences in the immune response to COVID-19 have previously been described, where higher levels of proinflammatory markers such as IL-8, IL-18, and CCL5 were found in the serum of males with COVID-19 than in females.^17^ However, how the host response to COVID-19 differs between males and females is not well understood. Identifying the host response biomarker profiles during hospitalization associated with sex-based differences in clinical outcomes may shed light on the different pathophysiological pathways underlying the differences observed between male and female patients in the severity and clinical course of COVID-19.

Therefore, we investigated whether circulating markers of inflammation, endothelial dysfunction, coagulation, organ injury, and adipokines were associated with the clinical parameters and outcomes of hospitalized COVID-19 patients. To do this, we performed a multicenter prospective study in which we collected plasma samples and clinical information from COVID-19 patients admitted to the general ward and ICU during the first year of the pandemic.

## Results

### Study cohort and design

Of 303 hospitalized COVID-19 patients admitted to the University Medical Center Groningen (UMCG) and Amphia hospital, 170 (56%) had severe COVID-19 requiring general ward care, whereas 133 (44%) were in critical condition and required invasive mechanical ventilation and organ support for multiple failing organs in the ICU (Figure S1). The baseline characteristics of the patients are shown in Table 1. The median age of patients with severe COVID-19 was 67 years (IQR 57–76), which was higher than that of critically ill COVID-19 patients admitted to the ICU (64, IQR 56–72) (Table 1). Seventy-one percent of the critical COVID-19 patients were male, whereas in the general ward, 55% of the patients were male (Table 1). The median BMI of critical COVID-19 was 28.1 (IQR 25.4–31.6) and was slightly higher than that of severe COVID-19 patients (27.0 IQR 24.3–30.7) (Table 1). As expected, critical COVID-19 patients experienced a greater number of complications, such as thromboembolic events (pulmonary embolism [28.6% vs. 2.9%], stroke [7.5% vs. 0.6%]), and acute kidney injury (45.9% vs. 6.5%) compared to severe COVID-19 patients. Additionally, COVID-19 patients admitted to the ICU often had respiratory co-infection or another infection leading to bacteriemia (20.3% vs. 0%). Patients with critical COVID-19 were hospitalized for significantly longer than those with severe COVID-19 (5 [4–9] vs. 22 [16–40] days).

**Table 1:** Demographic and clinical characteristics

Variable	Total COVID-19	A Severe COVID-19	B Critical COVID-19	*p*-value A vs. B
	*n* = 303	Ward *n* = 170	ICU *n* = 133	
**Demographics**				

Age (years)	66 (56–74)	67 (57–76)	64 (56–72)	0.021
Male, n (%)	188 (62)	93 (54.7)	95 (71.4)	0.003
BMI (kg/m^2^)	27.8 (24.7–30.8)	27.0 (24.3–30.7)	28.1 (25.4–31.6)	0.049

**Comorbidity**				

Pulmonary disease, n (%)	66 (22.8)	37 (21.8)	29 (21.8)	0.993
Chronic cardiac disease, n (%)	76 (25.1)	43 (25.3)	33 (25.3)	0.923
Chronic kidney disease, n (%)	27 (8.9)	18 (10.6)	9 (6.8)	0.247
Hypertension, n (%)	107 (35.3)	60 (35.3)	47 (35.3)	0.994
Diabetes, n (%)	63 (20.7)	38 (22.4)	25 (18.8)	0.449

**Clinical course**				

O2 need at hospital admission (L/min)	3 (0–5)	2 (0–4)	11 (3–15)	<0.001
Hospital length of stay, days	10 (5–21)	5 (4–9)	22 (16–40)	<0.001

**Treatment**				

Antiviral	53 (17.5)	35 (20.8)	18 (13.5)	0.118
Antibiotics	240 (79.2)	109 (64.1)	131 (98.5)	<0.001
Corticosteroids	121 (39.9)	55 (32.6)	66 (49.6)	0.002
Antifungal therapy	32 (10.6)	0 (0)	32 (24.0)	<0.001
Chloroquine	140 (46.2)	78 (45.9)	62 (46.6)	0.899

**ICU characteristics**				

APACHE II score	N/A	N/A	15 (12–19)	N/A
ICU length of stay, days	N/A	N/A	16 (9–29)	N/A
Duration of mechanical ventilation, days	N/A	N/A	13 (8–25)	N/A
PaO2/FiO2 after intubation	N/A	N/A	134 (101–178)	N/A
Prone ventilation	N/A	N/A	102 (76.7)	N/A
Tracheostomy	N/A	N/A	16 (12.0)	N/A
ECLS	N/A	N/A	8 (6.0)	N/A

**Complications**				

Pulmonary Embolism	43 (14.2)	5 (2.9)	38 (28.6)	<0.001
Pneumothorax	5 (1.7)	1 (0.6)	4 (3.0)	0.173
Stroke	11 (3.6)	1 (0.6)	10 (7.5)	0.001
Cardiac ischemia	8 (2.6)	5 (2.9)	3 (2.3)	0.999
Bacteremia∗	27 (8.9)	0 (0)	27 (20.3)	<0.001
Acute kidney injury∗∗	72 (23.8)	11 (6.5)	61 (45.9)	<0.001
Renal replacement therapy	25 (8.3)	0 (0)	25 (18.8)	<0.001
Liver dysfunction∗∗∗	97 (32.0)	26 (15.3)	71 (53.4)	<0.001
Death	–	–	42 (31.6)	–

Data are presented as median [iQR], or n and percentage. *p* values are calculated using Mann Whitney U, Chi-squared test or Fishers exact test. APACHE: acute physiology and chronic health evaluation, BMI: body mass index, ECLS: extra corporeal life support, ICU: intensive care unit, ∗positive blood cultures, ∗∗increase in serum creatinine by 26.5 μmol/L within 48 h, or >1.5 times baseline within the prior 7 days, ∗∗∗an increase in blood bilirubin, alanine transaminase or aspartate transaminase twice the upper limit of the normal range, n/a-not applicable.

### Differential host response biomarker profiles associated with COVID-19 severity

**Figure 1:**
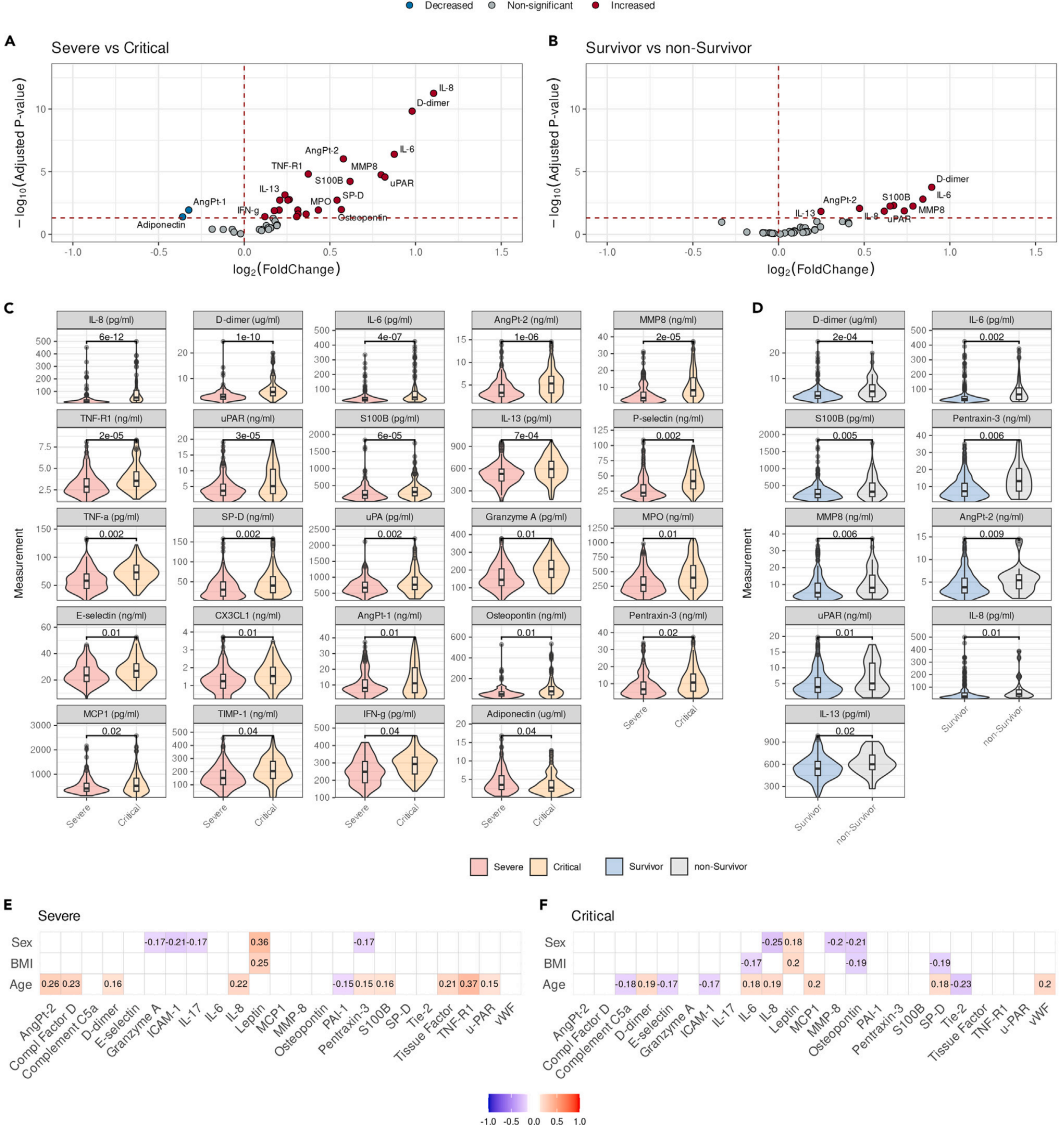
Host response markers associated with the severity and mortality of COVID-19 (A and B) Volcano plot visualizing the proteins associated with COVID-19 severity in severe (ward patients) versus critical (ICU patients) COVID-19 severity (A) and mortality (B). The x axis represents the difference between both conditions (Log2 Fold Change), while the y axis represents the adjusted *p* value. *p* values are adjusted using the Benjamini Hochberg correction. (C and D) Violin-plots visualizing the distribution of protein expression associated with COVID-19 severity (C) and mortality (D). The boxplot displayed inside the violin indicates the median with interquartile range (IQR). The x axis and colors represent the different groups basis on COVID-19 severity and mortality, while the y axis represents the protein expression. Benjamini Hochberg adjusted *p* values are visualized in each panel, ns = not significant. (E and F) Heatmap visual representation of the correlations between proteins expression and clinical parameters stratified by COVID-19 severity. The y axis represents the clinical variables, including sex, BMI, and age, while the x axis represents the investigated proteins. The colors in the heatmap indicate the strength of the observed correlations, with red indicating positive correlations and blue indicating negative correlations. Correlations coefficients with respect to sex can be interpreted as follows, negative values indicate increased expression in males and positive values increased expression in females.

We hypothesized that the levels of plasma host response markers, which may be affected by factors such as age, sex, and BMI, are related to the severity of COVID-19. To investigate this, we determined the plasma levels of various inflammatory cytokines, chemokines, adipokines, endothelial cells, coagulation markers, and organ injury markers using multiplex analyses (Luminex Corporation, Austin, USA). A list of the proteins analyzed and the details of their functions are available in Table S1. Preceding our differential expression analysis, we performed principal-component analysis (PCA) analysis to assess the potential influence of clinical features on our results. This analysis revealed minor differences between COVID-19 patients admitted to the ward or ICU; however, the two groups did not segregate into distinct clusters (Figure S3A). Additionally, we observed no clear clustering of male and female patients (Figure S3B). In contrast, distinct clusters were observed when comparing patients admitted to the UMCG or Amphia hospital, which might have been attributable to the sampling logistics, transport times to the lab, and/or differing manufacturers and/or batches of tubes used for blood collection (Figure S3C). To account for these site differences, we applied a normalization correction based on hospital of admission, which led to a homogeneous cluster (Figure S3D). Among the proteins analyzed, we identified 22 proteins with elevated concentrations in patients with critical compared to those with severe COVID-19, whereas Angpt-1 and adiponectin levels were lower in patients with critical COVID-19 than in those with severe COVID-19 (Figures 1A and 1C; Table S2). Next, we determined whether the host response protein profile differed between critical COVID-19 survivors and non-survivors. We identified higher levels of D-dimer, IL-6, IL-8, IL-13, Angpt-2, Pentraxin-3, S100B, MMP-8, and u-PAR in the plasma of critical COVID-19 non-survivors than in that of survivors. (Figures 1B and 1D; Table S3).

To investigate whether the variation in biomarker protein levels between patients with severe and critical COVID-19 could be attributed to differences in sex, BMI, and age, a correlation analysis was performed for these variables in both patient groups (Figures 1E, 1F, S4, and S5). In patients with severe and critical COVID-19, D-dimer, and IL-8 were correlated with age, while leptin levels were positively correlated with BMI and sex, indicating increased levels in males. In addition, granzyme A, ICAM-1, IL-17 and pentraxin-3 levels are higher in severe females, while IL-8, MMP-8 and osteopontin were increased in critical females. Moreover, IL-6 and osteopontin were correlated with BMI in critical COVID-19 patients in contrast to severe COVID-19 patients. IL-6 and SP-D are correlated with both BMI and age, while complement C5a, E-selectin, ICAM-1, MCP-1, Tie-2, and vWF levels were associated with age in patients with critical, but not severe COVID-19. Additionally, Angpt-2, complement factor D, PAI-1, pentraxtin-3, S100B, tissue factor, TNF-R1, and u-PAR levels were associated with age in patients with severe, but not critical COVID-19. These data suggest that the correlation between these proteins and age is confounded by COVID-19 severity and the role of these host response proteins in mediating the critical disease course.

### Sex differences in the levels of host response markers were dependent on COVID-19 severity

We observed that more male patients (71%) were admitted to the ICU with critical COVID-19, which is consistent with previous reports.^9^ However, we did not observe differences in patient demographic parameters or pre-existing comorbidities between males and females among COVID-19 patients admitted to either the ward or the ICU (Table 2). Additionally, the ICU clinical course and characteristics were similar between male and female patients with critical COVID-19. Moreover, the incidence of complications during hospitalization was similar between male and female severe COVID-19, except that male patients had a higher incidence of liver dysfunction (21.5% vs. 7.8%, *p* = 0.013). In critically ill COVID-19 patients, the incidence of complications during ICU stay was similar between the sexes, except for a higher incidence of male patients needing renal replacement therapy (RRT) (23% vs. 7.9%, *p* = 0.042). Therefore, although the incidence of acute kidney injury (AKI) was similar between male and female critical COVID-19 patients, the severity of AKI was more severe in males because they required RRT, whereas females did not.

**Table 2:** Demographic and clinical characteristics, subdivided by sex

Variable	A Ward Males	B Ward Female	C ICU Males	D ICU Females	*p* value A vs. B	*p* value C vs. D
	*n* = 93	*n* = 77	*n* = 95	*n* = 38		
**Demographics**						

Age (years)	67 (58–77)	67 (55–77)	65 (55–72)	63 (58–71)	0.591	0.905
BMI (kg/m^2^)	26.3 (24.1–30.0)	28.0 (24.4–31.8)	27.8 (24.8–30.6)	29.2 (26.1–32.4)	0.126	0.059

**Comorbidity**						

Pulmonary disease, n (%)	15 (16.1)	22 (28.6)	18 (18.9)	11 (28.9)	0.050	0.207
Chronic cardiac disease, n (%)	25 (26.9)	18 (23.4)	26 (27.4)	7 (18.4)	0.601	0.280
Chronic kidney disease, n (%)	10 (10.8)	8 (10.4)	6 (6.3)	3 (7.9)	0.939	0.743
Hypertension, n (%)	36 (38.7)	24 (31.2)	36 (37.9)	11 (28.9)	0.306	0.329
Diabetes, n (%)	19 (20.4)	19 (24.7)	17 (17.9)	8 (21.1)	0.508	0.674

**Clinical course**						

Symptom days before hospital	10 (6–12)	9 (5–12)	8 (7–11)	7 (6–10)	0.237	0.093
O2 hospital admission (L/min)	2 (0–4)	2 (0–4)	14 (3–15)	6 (2–15)	0.550	0.537
Hospital length of stay, days	5 (4–9)	5 (4–9)	21 (15–42)	24 (16–38)	0.814	0.504

**Treatment**						

Antiviral	19 (20.4)	16 (21.3)	17 (18.5)	1 (2.6)	0.886	0.017
Antibiotics	61 (65.6)	48 (63.2)	93 (99)	38 (100)	0.750	0.523
Corticosteroids	28 (30.1)	27 (35.1)	48 (50.5)	18 (47.4)	0.492	0.742
Antifungal therapy	0 (0)	0 (0)	23 (24.7)	9 (23.7)	1.000	0.899
Chloroquine	48 (51.6)	30 (39.0)	47 (49.5)	15 (39.5)	0.099	0.296

**ICU characteristics**						

APACHE II score	N/A	N/A	15 (12–18)	15 (12–22)	–	0.403
ICU length of stay, days	N/A	N/A	15 (9–24)	16 (10–28)	–	0.558
Duration MV, days	N/A	N/A	13 (8–24)	15 (8–25)	–	0.514
PaO2/FiO2 after intubation	N/A	N/A	135 (112–179)	122 (83–177)	–	0.232
Prone ventilation	N/A	N/A	70 (76.1)	32 (84.2)	–	0.305
Tracheostomy	N/A	N/A	11 (12.0)	5 (13.2)	–	0.999
ECLS	N/A	N/A	5 (5.4)	3 (7.9)	–	0.691

**Complications**						

Pulmonary Embolism	2 (2.2)	3 (3.9)	31 (32.6)	7 (18.4)	0.659	0.101
Pneumothorax	1 (1.1)	0 (0)	2 (2.1)	2 (5.3)	0.999	0.322
Stroke	1 (1.1)	0 (0)	5 (5.3)	5 (13.2)	0.999	0.148
Cardiac arrhythmia	5 (5.4)	2 (2.6)	22 (23.2)	8 (21.1)	0.458	0.793
Cardiac ischemia	3 (3.2)	2 (2.6)	3 (3.2)	0 (0)	0.999	0.557
Bacteremia∗	0 (0)	0 (0)	19 (20.0)	8 (21.1)	1.000	0.892
Acute kidney injury∗∗	9 (9.7)	2 (2.6)	45 (47.4)	16 (42.1)	0.114	0.582
Renal replacement therapy	0 (0)	0 (0)	22 (23.2)	3 (7.9)	1.000	0.042
Liver dysfunction∗∗∗	20 (21.5)	6 (7.8)	51 (53.7)	20 (52.6)	0.013	0.912
Death	–	–	29 (30.5)	13 (34.2)	–	0.680

Data are presented as median [iQR], or n and percentage. *p* values are calculated using Mann Whitney U, Chi-squared test or Fishers exact test. APACHE: acute physiology and chronic health evaluation, BMI: body mass index, ECLS: extra corporeal life support, ICU: intensive care unit, ∗positive blood cultures, ∗∗increase in serum creatinine by 26.5 μmol/L within 48 h, or >1.5 times baseline within the prior 7 days, ∗∗∗an increase in blood bilirubin, alanine transaminase or aspartate transaminase twice the upper limit of the normal range, MV- mechanical ventilation, n/a- Not applicable.

**Figure 2:**
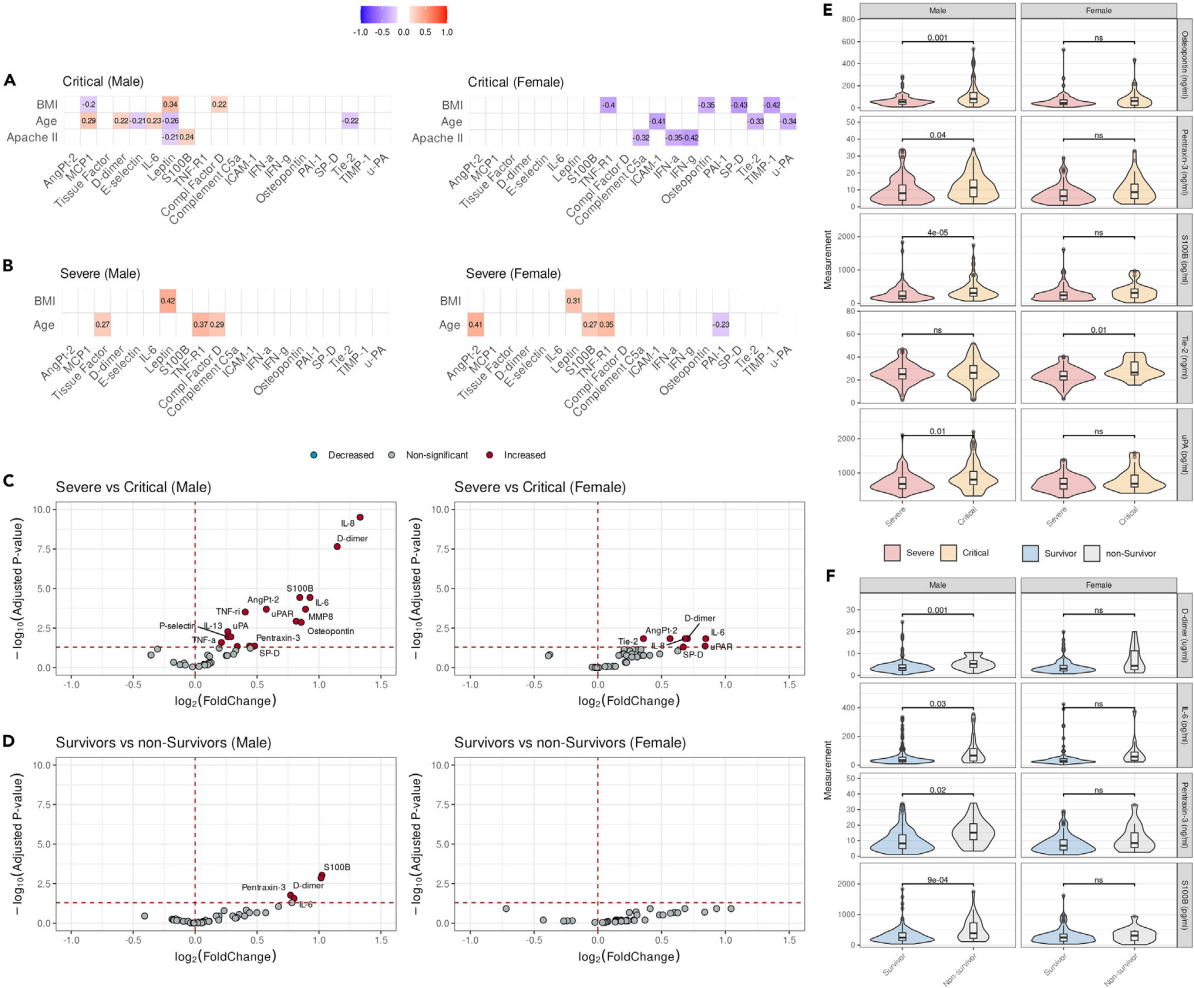
Sex-specific host response markers associated with the severity and mortality of COVID-19 (A and B) Heatmap visual representation of the sex-specific correlations between proteins expression and clinical parameters stratified by COVID-19 severity. The y axis visualizes the clinical variables, including Apache II, BMI, and age, while the x-as represents the investigated proteins. The colors in the heatmap indicate the strength of the observed correlations, with red indicating positive correlations and blue indicating negative correlations. (C and D) Volcano plot visualizing the proteins associated with COVID-19 severity (top) and mortality (bottom) in males (left side) and females (right side). The x axis represents the difference between both conditions (log2 Fold Change), while the y axis represents the adjusted *p* value. *p* values are adjusted using the Benjamini Hochberg correction. (E and F) Violin boxplots visualizing the distribution of sex specific protein expression associated with disease severity and mortality in males and females. The y axis represents the protein expression. The boxplot displayed inside the violin indicates the median with interquartile range (IQR). Benjamini Hochberg adjusted *p* values are visualized in each panel, ns = not significant.

After observing the correlation of proteins with age, BMI, and sex in patients with severe and critical COVID-19, we explored whether these correlations were sex-specific and assessed whether any of the proteins were correlated with the APACHE II score, a classification of critical illness severity.^18^ For this sub analysis, we focused on critical COVID-19 patients, as the APACHE II score only applies to patients admitted to the ICU and performed correlation analyses in a sex-stratified manner (Figures 2A, S6, and S7).

Interestingly, while in the overall analysis, we did not observe any significant correlation between the values of the proteins and the APACHE II score, we observed a moderate negative correlation with IFN-a, IFN-g, and complement C5a in females and a moderate positive correlation with S100B and negative correlation with leptin in males. Four proteins (SP-D, TIMP-1, TNF-R1, and osteopontin) showed a moderate correlation with BMI, specifically in females, whereas osteopontin was also observed in the overall analysis, but not in males. Complement factor D was specifically correlated with BMI in males, while leptin was also correlated in the overall analysis, but not in critically ill females. Other noteworthy sex-specific correlations observed in severe COVID-19 patients are leptin, which is correlated with BMI, while TNF-R1 is correlated with age in both severe males and females (Figure 2B). Angpt-2, S100B and PAI-1 are correlated with age in females, while tissue factor and complement factor D were correlated with age in males. These results highlight that the levels of proteins are affected by BMI and age in a sex-stratified manner and that some proteins correlate with severity differently in males and females.

As a result of these sex-specific correlations, we aimed to identify proteins that are differentially regulated based on disease severity in males and females. To achieve this, a sex-stratified differential abundance analysis including all covariates was performed. We identified 16 plasma proteins that were present at higher concentrations in critically ill male COVID-19 patients than in severe COVID-19 patients admitted to the general ward (Figure 2C left; Table S4). In female COVID-19 patients, we identified seven circulating plasma proteins with higher concentrations in those with critical COVID-19 than in those with severe COVID-19 (Figure 2C right; Table S5). Among the identified proteins, osteopontin, pentraxin-3, S100B, and u-PA levels were significantly different only in males, whereas Tie-2 levels were significantly different only in the plasma of females (Figure 2E). To determine whether these differences could be observed in clinical practice, we assessed the differences in protein concentrations with and without correction for covariates (Figures S8 and S9).

Furthermore, we performed a similar analysis comparing the circulating protein levels in males who died versus those who survived critical COVID-19. We identified four significantly upregulated proteins, S100B, D-dimer, pentraxin-3, and IL-6 (Figure 2D left; Table S6). We did not identify any significantly different circulating proteins in female critical COVID-19 patients who died in the ICU compared with survivors (Figure 2D right; Table S7). Our results verified that D-dimer, IL-6, pentraxin-3 and S100B were significantly higher in male patients, but not in females (Figure 2F; Table S7).

In summary, we identified several plasma markers associated with critical COVID-19 in both males and females. Moreover, we identified markers specifically associated with mortality in critically ill male patients with COVID-19.

## Discussion

Sex differences in COVID-19 severity and mortality rates were consistent in most countries. Globally reported COVID-19 epidemiological data disaggregated by sex have found that SARS-CoV-2 infection rates appear to be similar between males and females.^19^^,^^20^ However, men develop more severe disease and are more likely to die than women.^7^ In this study, through sex-stratified analyses, we identified shared circulating protein signatures between males and females in critical COVID-19 cases, as well as sex-specific proteins unique to each sex. Increased levels of D-dimer, IL-6, pentraxin-3, and S100B were specifically associated with mortality in male critical COVID-19 patients only. Interestingly, increased levels of circulating soluble Tie-2 were observed in females with critical COVID-19. These findings suggest that sex-specific mechanisms may partially contribute to the observed variations in severity between males and females. Currently, all critically ill COVID-19 patients are treated in the same manner, which may not always be advantageous. Our findings have significant implications and suggest the need for treatment strategies to be customized for equal effectiveness in both sexes. This may involve employing different approaches, based on the respective mechanisms involved.

In contrast to previous studies conducted to identify biomarkers for COVID-19 severity, our study utilized a sex-specific approach. Moreover, some studies compared the protein levels of males and females against controls, but did not investigate sex-specific effects by performing a sex-stratified analysis.^17^ In our study, using a sex-stratified approach, we identified proteins whose levels were specifically associated with COVID-19 severity in male and females. Interestingly, we observed that IFN-a, IFN-g, and complement C5a were only correlated with APACHE II scores in females with critical COVID-19, but not in males or in the overall comparison. Interferon responses are known to play a critical role in antiviral immunity and may contribute to overall differences in responses between males and females.^21^ Consistent with the negative correlation of IFN proteins observed in our study, it has been shown that the *TLR7* gene that induces type I interferon response is downregulated in males compared to females in severe cases of COVID-19.^22^ These results highlight the importance of sex-stratified analyses.

Several hypotheses have been proposed to explain why males are more susceptible to COVID-19. Sex-associated differences in the expression of ACE2 receptor and TMPRSS2 have been identified; however, to date, only conflicting data have been published.^19^^,^^23^^,^^24^ Sex differences in immune responses have been reported long before the COVID-19 pandemic^25^^,^^26^^,^^27^ and are more likely to explain sex-specific responses to COVID-19. In line with this, several studies investigating inflammatory markers in hospitalized patients have been published.^17^^,^^28^^,^^29^^,^^30^ The levels of proinflammatory cytokines and chemokines (IL-8, IL-18, CCL5) were higher in males with severe COVID-19, and the T cell response was less robust than that in females.^17^ This may provide women with effective innate immune response mechanisms that facilitate rapid viral clearance. The impaired antiviral response in males may result in an enhanced susceptibility to severe COVID-19.

We identified several circulating proteins that were increased in both male and female critically ill COVID-19 patients. These markers reflect a pro-inflammatory phenotype (IL-6 and IL-8), endothelial dysfunction (Angpt-2), alveolar epithelial injury (SP-D), and soluble u-PAR is known to drive renal injury. However, we also identified circulating proteins that were specifically associated with critical COVID-19 in males only, which might drive the increased susceptibility and more severe symptoms observed in male patients. IL-13 attracts eosinophils and M2 macrophages to the lungs and is associated with the proliferation of smooth muscle cells, mucus generation, and fibrosis.^31^^,^^32^ D-dimer, a byproduct of fibrin breakdown, is an indirect indicator of thrombotic activity, and is therefore used clinically to identify elevated hypercoagulable states. Higher admission and peak D-dimer levels appear to be linked to deteriorating clinical outcomes, critical COVID-19 symptoms and mortality.^33^ In line with our findings, D-dimer has recently been proposed as a useful prognostic indicator for male COVID-19 infection patients.^34^ Corroborating our results, circulating IL-6 levels were higher in men than women with severe COVID-19.^28^ However, a recent meta-analysis found that monitoring IL-6 levels alone was not associated with mortality.^35^ Upon activation by pro-inflammatory cytokines, such as TNF-α and IL-1β, endothelial cells express CX3CL1 (fractalkine) and P-selectin. Elevated levels indicate endothelial activation, leading to dysfunction. Coupled with platelet activation, this process contributes to the development of thrombosis. However, sex-specific differences in this context have not been reported in the COVID-19 setting. Increased plasma levels of the Tie-2 receptor antagonist angiopoietin-2 (Angpt-2) in severely ill COVID-19 patients have been reported previously^36^^,^^37^^,^^38^^,^^39^ and were associated with ICU admission^39^ and the development of acute kidney injury.^40^ Moreover, soluble Tie-2 has been found to be associated with COVID-19 severity.^36^ We found elevated levels of Angpt-2 in critical COVID-19 patients but higher levels of soluble Tie-2 only in female patients with critical COVID-19. Sex-specific variations in circulating levels of soluble Tie-2 or Angpt-2 in COVID-19 patients have not been reported or studied in sepsis despite extensive research on the Tie-2-angiopoietin system in sepsis. Therefore, our study is the first to identify a role for soluble Tie-2 specifically in females with critical COVID-19. Mechanistically, soluble Tie-2 has been shown to bind to circulating angiopoietins and inhibit angiopoietin-mediated Tie-2 phosphorylation and anti-apoptotic signaling.^41^ Therefore, it may function as a protective mechanism by sequestering circulating angiopoietin-2. These results may explain the differences in favorable outcomes between female and male patients, but larger cohort studies are necessary to confirm this observation.

### Conclusion

In conclusion, our study identified distinct sex-specific plasma protein signatures linked to the severity and mortality of COVID-19. The observed hyper-inflammatory and thrombotic phenotypes in male patients suggest a potential association between heightened susceptibility to critical COVID-19 and increased mortality. These findings underscore the need for in-depth investigations into the roles of these circulating proteins in both sexes and their implications for the severity and prognosis of critical illnesses caused by other etiologies.

### Limitations of the study

A strength of this cross-sectional study is the inclusion of a substantial number of patients with differing severities from two different medical centers. Most previous studies also did not account for age, which is hypothesized to affect the host response to SARS-CoV-2 more than sex-specific differences. Our data, corrected for age, BMI, comorbidities, and steroid use, revealed sex-specific plasma profiles. However, our study has some limitations that must be considered. Since clinical data and blood samples were collected during the pandemic, we were forced to rely solely on the patient’s BMI as an easy and quick method to calculate body fat mass. Despite our best efforts, collecting blood samples at admission was not always feasible due to patient reallocation from hospitals and ICUs elsewhere in the Netherlands. We included samples from patients who were promptly collected after ward hospitalization or ICU admission. However, we cannot exclude the possibility that delays in plasma collection may have influenced the outcomes of this study. Despite the inclusion of a sizable patient cohort from two hospitals, the limited number of female ICU admissions and deaths from critical COVID-19 reduced the analytical power for a detailed mortality analysis. It is also important to recognize that biological variables cannot account for all sex-specific differences in COVID-19 severity. Although potentially significant, environmental, and social variables were not investigated in this study. Men’s higher susceptibility to smoking, compromising the immune system, and elevating the risk of severe COVID-19, along with a greater likelihood of concomitant conditions such as diabetes and hypertension, further amplifies the risk of severe COVID-19 in men than in women. Despite this, we found no discernible variation in the prevalence of underlying comorbidities between males and females in our study. Hence, it is unlikely that the observed variations in plasma marker levels could be attributed to comorbidities.

## Resource availability

### Lead contact

Further information and requests for resources should be directed to and will be fulfilled by the lead contact Jill Moser (j.moser@umcg.nl).

### Material availability

This study did not generate new unique reagents.

### Data and code availability

• Data: The data used for this study has been deposited into Medeley data and is publicly available as of the date of publication. The DOI is listed in the key resources table.
• Code: This manuscript does not report original code.
• All other requests: Any additional information required to reanalyze the data reported in this paper is available from the lead contact upon request.

## Acknowledgments

We would like to thank Karin Koerts and Thijs Heerink from the Department of Laboratory Medicine-UMCG, Johan Bijzet from the Department of Rheumatology & Clinical Immunology-UMCG, and Rianne Jongman from the Department of Anesthesiology-UMCG, for providing excellent technical support. In addition, we would like to acknowledge Judith Emmen from the Amphia hospital and Hildegard Franke from the UMCG. This work was supported by 10.13039/501100001826ZonMw (project numbers: 10430012010006 & 10430012010002).

## Author contributions

J.M., V.K., P.V., P.H., I.R.Z., A.M.K., and S.S., were responsible for the study design. P.V., S.S., A.M.K., and M.v.M. were responsible for sample collection. A.W.F., J.M., N.K., I.R.P., and A.M.K. were responsible for data collection. N.K., A.W.F., I.R.P., K.L., J.M., and V.K. were responsible for data analysis. The first draft of the manuscript was written by N.K., A.W.F., I.R.P., A.M.K., M.J.G., J.M., and V.K. which was subsequently revised based on the input from all authors. All authors read and approved the final version of the manuscript.

## Declaration of interests

The authors declare no competing interests.

## STAR★Methods

### Key resources table

**Table:** 

REAGENT or RESOURCE	SOURCE	IDENTIFIER
**Biological samples**		

Human Patient Plasma samples	UMCG/Amphia	N/A

**Critical commercial assays**		

Human Luminex xMAP multiplex assays	Luminex	https://www.thermofisher.com/

**Deposited data**		

Proteomic data	Mendeley data	https://doi.org/10.17632/34kn7422t5.1

**Software and algorithms**		

R version (4.2)	R development Core Team	http://www.R-project.org
ggplot2 R package (v3.5.1)	CRAN (https://cran.r-project.org)	https://doi.org/10.32614/CRAN.package.ggplot2
Factoextra R package (v1.0.7)	CRAN (https://cran.r-project.org)	https://doi.org/10.32614/CRAN.package.factoextra
Limma R package (v3.6.9)	CRAN (https://cran.r-project.org)	https://doi.org/10.1093/nar/gkv007
Pwr R package (v1.3.0)	CRAN (https://cran.r-project.org)	https://doi.org/10.32614/CRAN.package.pwr
xPONENT v4.2 software (Luminex)	Thermofisher	https://www.thermofisher.com/

### Experimental model and study participant details

For this study, we collected plasma samples and clinical data from COVID-19 patients admitted to two different hospitals in The Netherlands, the University Medical Center Groningen (UMCG) and Amphia Hospital in Breda. The inclusion periods were the 6^th^ of March 2020 to the 3^rd^ of April 2020 (Amphia Hospital; 1^st^ wave), the 24^th^ of April to the 6^th^ of June 2020 (UMCG; 1^st^ wave), and the 28^th^ of September 2020 to the 3^rd^ of December 2020 (UMCG; 2^nd^wave). The term ‘Severe COVID-19’ was used to designate patients admitted to the general ward and the term ‘critical COVID-19’ was used to designate patients admitted to the ICU. Plasma was collected from severe COVID-19 patients within 48h after admission. However, for critical COVID-19 patients, blood collection at ICU admission was not always possible due to patients transferring from other ICUs. Therefore, samples from these patients were collected as soon as possible after ICU admission and within 72h.

#### Severe and critical COVID-19 patients

This study included adult COVID-19 patients hospitalized during the first two infection waves in the Netherlands and admitted to the UMCG and Amphia hospitals (Figure S1). SARS-CoV-2 infection was determined by RT-PCR of oropharyngeal and nasopharyngeal swabs. Patients were treated according to the local COVID-19 treatment protocol. Routine ICU care comprised high-dose anticoagulation with low-molecular-weight heparin (LWMH) (87IE/kg twice daily) and selective digestive tract decontamination. Chloroquine was initially used as part of the standard ward and ICU therapy during the first COVID-19 wave, until the Netherlands National Institute for Public Health and Environment advised against its use at the end of March 2020. All 2nd wave patients who were admitted to the hospital starting in July 2020 received dexamethasone 6 mg daily if they required supplemental oxygen therapy, and some patients also received remdesivir if their symptoms lasted less than 10 days. This study excluded patients who died in the general ward because of treatment restrictions made in accordance with the patient's wishes. None of the COVID-19 hospitalized patients received the SARS-CoV-2 vaccine. The need for informed consent was waived (UMCG METc 2020/492 and Medical Research Ethics Committees United W20.248/Central Research Committee Amphia N2020-0380) because the analyses were performed using residual plasma samples needed for clinical purposes.

### Method details

Residual heparinized plasma was collected from all hospitalized patients directly after routine analysis and stored at -80^o^C until biomarker analysis. Severe and critical COVID-19 patients included in our cohort were admitted to either the general ward or ICU approximately 8-10 days after the initial COVID-19 symptoms. Multi-analyte profiling of plasma was performed using custom-made Human Luminex xMAP multiplex assays (R&D Systems, Abingdon, UK) according to the manufacturer’s instructions and read on a Luminex 200 instrument (Luminex, Austin, TX, USA). Data analysis was performed using the xPONENT v4.2 software (Luminex). A list of the analytes is provided in Table S1. Markers of inflammation, adipokines, endothelial dysfunction, coagulation, and organ injury were also included. Concentration values below the lower limit of detection were set to the lowest value that could be accurately determined; if the upper limit of detection was reached, these values were set to the upper limit of quantification. Most values for IL-2 were below the limit of detection, whereas those for CXCL10 were above the detection limit; therefore, these proteins were excluded from further analysis. We previously showed that visfatin levels were higher in critical COVID-19 patients than in severe and mild COVID-19 patients,^42^ yet many of the samples were below the detection limit; therefore, we excluded visfatin from this analysis. Inter-assay variation was monitored using three of the seven calibration line points made on the first day of measurement, aliquoted, and stored at -80^o^C. These three aliquots were thawed and used as internal controls each day the analysis was performed and the analyte coefficient of variation (CV) was calculated (Figure S2).

#### Data collection

Patient clinical and demographic information were extracted from the electronic medical records of hospitalized individuals. Clinical information included age, sex, body mass index (BMI), medical history, and the clinical course of COVID-19 during hospital admission. A modified version of the World Health Organization (WHO) electronic Case Report Form (eCRF)^43^ was used to capture data in the Clinical Database Infrastructure Research Electronic Data Capture (REDCap).^44^ Body mass index (BMI) was used to estimate body fat mass and was defined as a person's weight in kilograms divided by the square of their height in meters (kg/m^2^).

### Quantification and statistical analysis

Statistical analyses and data processing were conducted using the R software (version 4.2). Continuous variables were expressed as median and interquartile range (IQR) values and subsequently assessed for significance using the Mann-Whitney U test and Kruskal-Wallis test. Categorical and dichotomous variables were expressed as frequencies and percentages and assessed using Chi-squared test and Fisher's exact tests. To evaluate correlations between our clinical variables and protein expression levels we utilized the spearman’s correlation test using the residuals of a standard linear model in which we corrected protein expression levels for hospital center. This approach allows to control for covariates by removing their influence on protein expression, Moreover, we used the R package “pwr” (v1.3.0) to conduct a post-hoc power analysis to ensure the study was sufficient powered to detect meaningful effects. We assumed a significance level of 0.05 and a moderate effect size of 0.5 log2 fold change. While the main analysis was adequately powered, the sex-specific analysis, especially the female’s specific analysis did not reach the desired level of 80% power threshold. Indicating results should be considered exploratory and therefore be interpreted with caution. The results are reported as volcano plots and box plots generated using the ggplot2 package(v3.5.1).^45^

#### Principal component analysis

Principal component analysis (PCA) variables were log2 transformed, scaled, and centered using the prompt function in R, and visualized using the “factoextra” package (v1.0.7). PCA was performed for all protein measurements from all patients. This analysis revealed minor differences between COVID-19 patients admitted either to the ward or ICU, although the two groups did not separate into distinct clusters (Figure S3A). We observed no clear clustering of male and female patients (Figure S3B). In contrast, separate clusters (batches) were observed when comparing patients admitted to the UMCG or Amphia hospital which might have been attributable to the logistics of sampling, transport times to the lab, and/or differing manufacturer and/or batch of tubes used for blood collection (Figure S3C). Therefore, we corrected for these hospital center differences using a standard linear model normalization correction for the hospital of admission, which led to a homogenous cluster (Figure S3D).

#### Differential abundance analysis

Cross-sectional differential abundance analyses were performed by applying robust linear regression using a generalized linear model (GLM) and additionally the R package limma(v3.6.9).^46^ Protein measurements were log2 transformed, and linear models were independently fitted for each protein, including age, BMI, sex, hospital center, diabetes, and corticosteroid administration as covariates. We estimated the Variance inflation Factor (VIF) score to assess the multicollinearity among all variables used in our linear models. Scores below 3 were considered to indicate low multicollinearity and allowed us to confidently include all relevant variables in our model without introducing instability in the parameter estimates. Lastly, results from the differential abundance analyses were corrected for multiple testing using the Benjamini-Hochberg method, and a false discovery rate (FDR) < 0.05 was considered statistically significant.

## Supplemental information

Document S1. Figures S1–S9

Table S1. Analytes measured in this study and associated function, related to Figure 1

Table S2. Results of DE Analysis Severity, related to Figure 1A

Table S3. Results of DE Analysis Mortality, related to Figure 1B

Table S4. Results of DE Analysis Severity (Male), related to Figure 2C

Table S5. Results of DE Analysis Severity (Female), related to Figure 2C

Table S6. Results of DE Analysis Mortality (Male), related to Figure 2D

Table S7. Results of DE Analysis Mortality (Female), related to Figure 2D
