# Supplementary material for: Delineating sex-specific circulating host response signatures associated with COVID-19 severity and mortality
Source: iScience. 2024 Oct 11;27(11):111150. doi: 10.1016/j.isci.2024.111150 (PMC11539596; doi:10.1016/j.isci.2024.111150)
Supplement: Document S1. Figures S1–S9 [file mmc1.pdf]

iScience, Volume *27*

Supplemental information

Delineating sex-specific circulating host response signatures associated with COVID-19 severity and mortality

Nick Keur, Antine W. Flikweert, Isis Ricaño-Ponce, Anneke C. Muller Kobold, Simone van der Sar-van der Brugge, Izabela A. Rodenhuis-Zybert, Kieu T.T. Le, Matijs van Meurs, Marco J. Grootenboers, Peter H.J. van der Voort, Peter Heeringa, Vinod Kumar, and Jill Moser

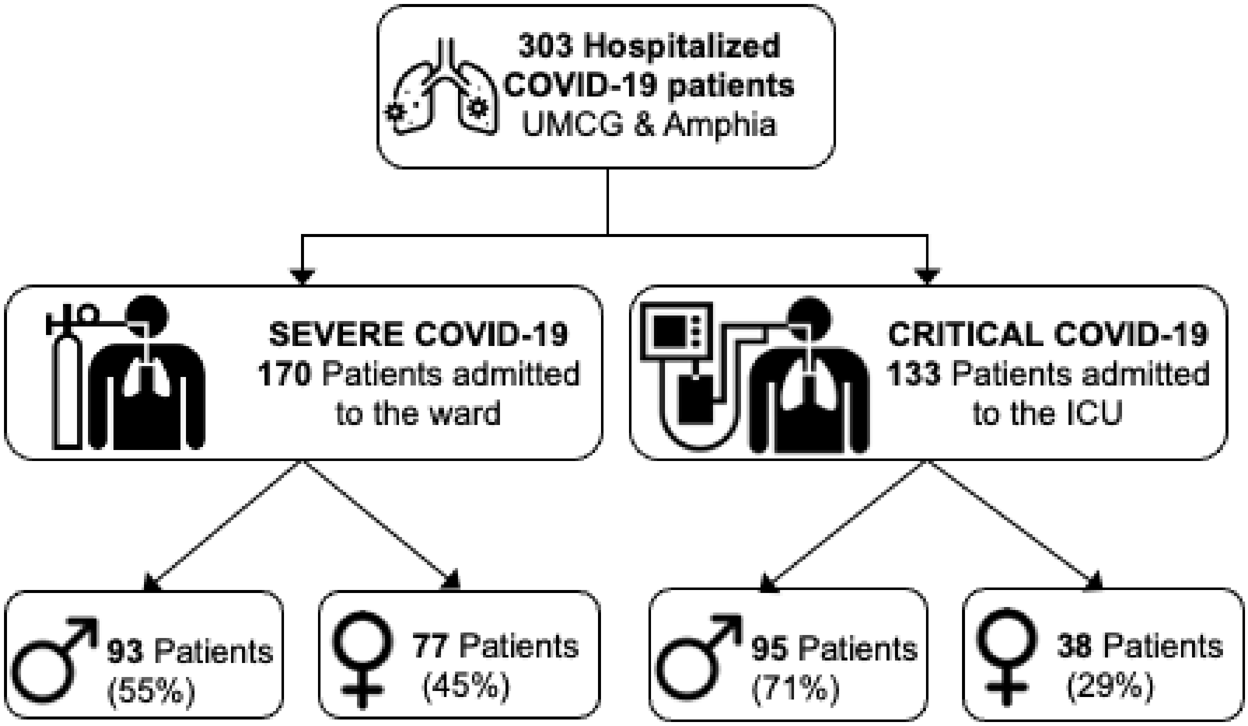

Supplemental figure 1

Overview of the study cohort and inclusion of patients.

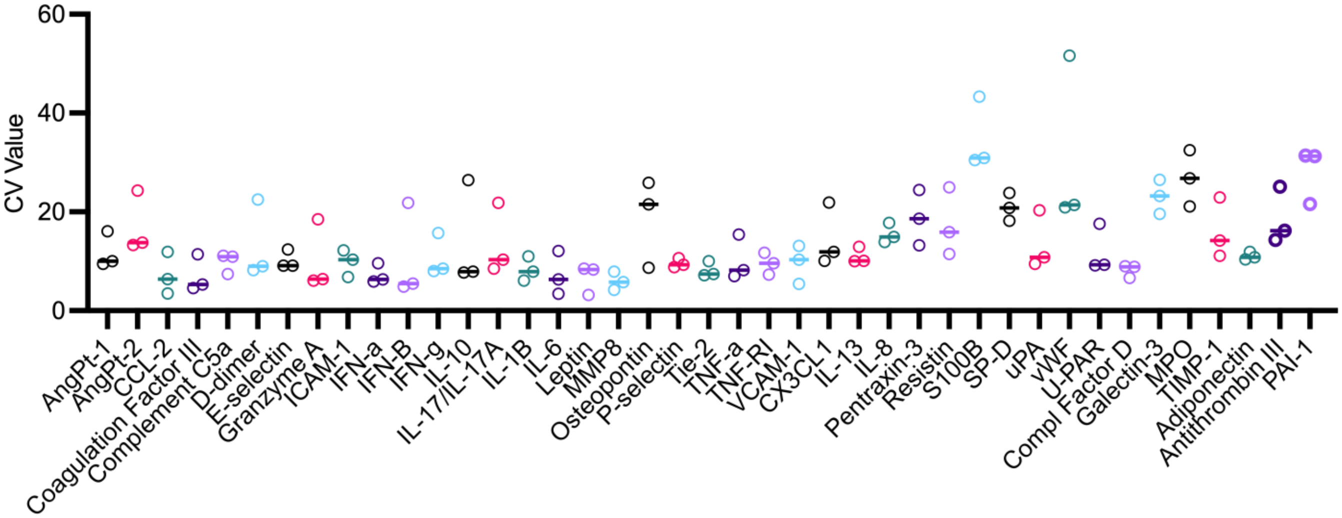

Supplemental figure 2

Overview of the standard of coefficient of variation for the Luminex assays

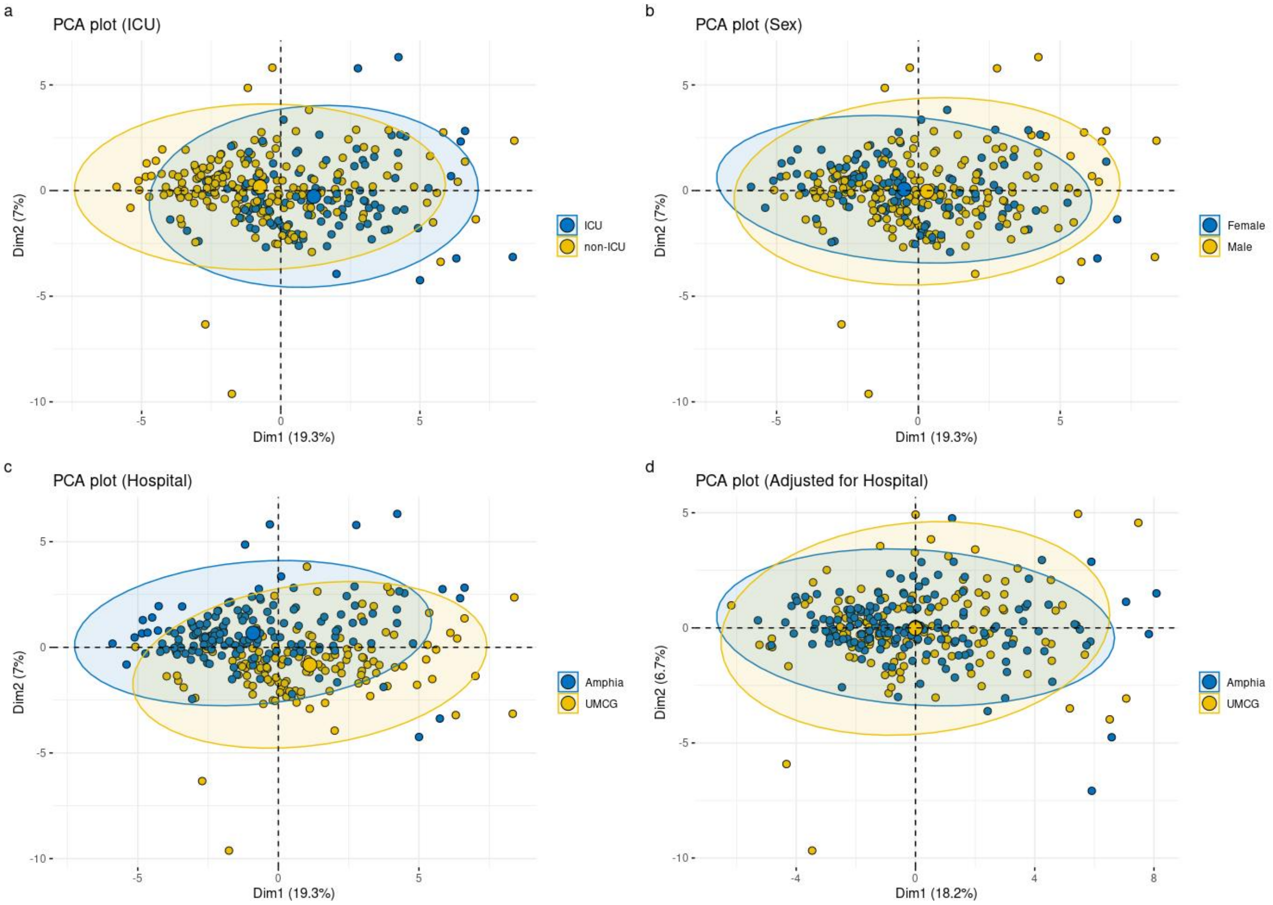

Supplemental Figure 3:

Principal component analysis to visualize demographics in reduced space. (a) ICU vs Ward (b) Male vs Female (c) Admission site, Amphia vs UMCG (d) Site adjusted Values, Amphia vs UMCG. Figures show the first two principal components, with demographics marked in different colours. Each point represents a single patient.

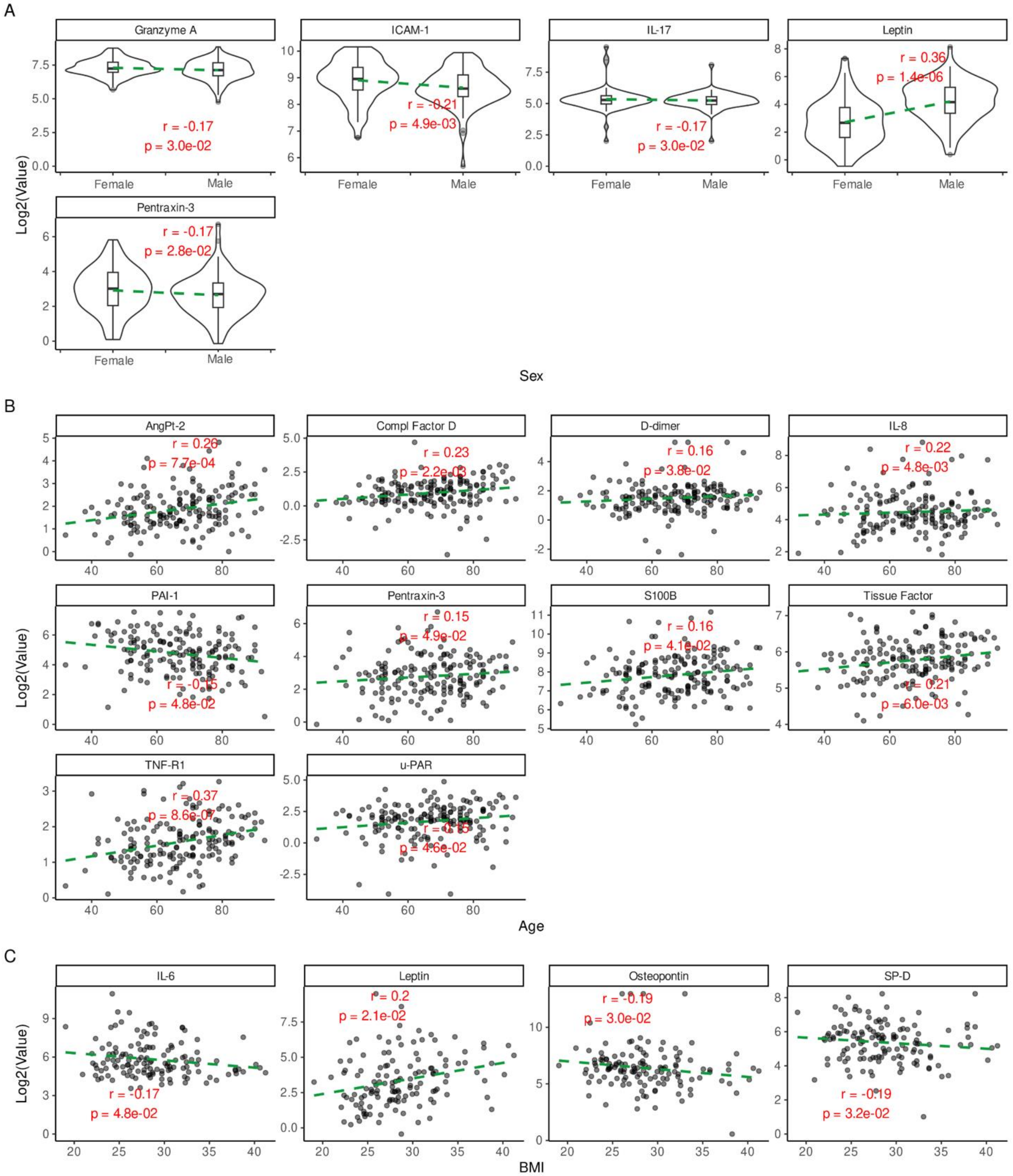

Supplemental Figure 4 : Correlations between Clinical variables and protein levels (Severe). Overview of proteins with significant correlations with clinical parameters. **(A)** Violin-plots visualizing proteins associated with sex in severe COVID-19 patients. The violin plot shows the log2 data distribution of the protein expression data and the boxplot displayed inside the violin indicates the median with interquartile range (IQR). (**B**) Scatter plots visualizing the significant correlation between protein levels and age in severe COVID-19 patients. **(C)** Point scatter plots visualizing the significant correlation between protein levels and BMI in severe COVID-19 patients. In each plot the x-axis represent the clinical parameter of interest, while the y-axis represents the protein levels after log2 transformation.

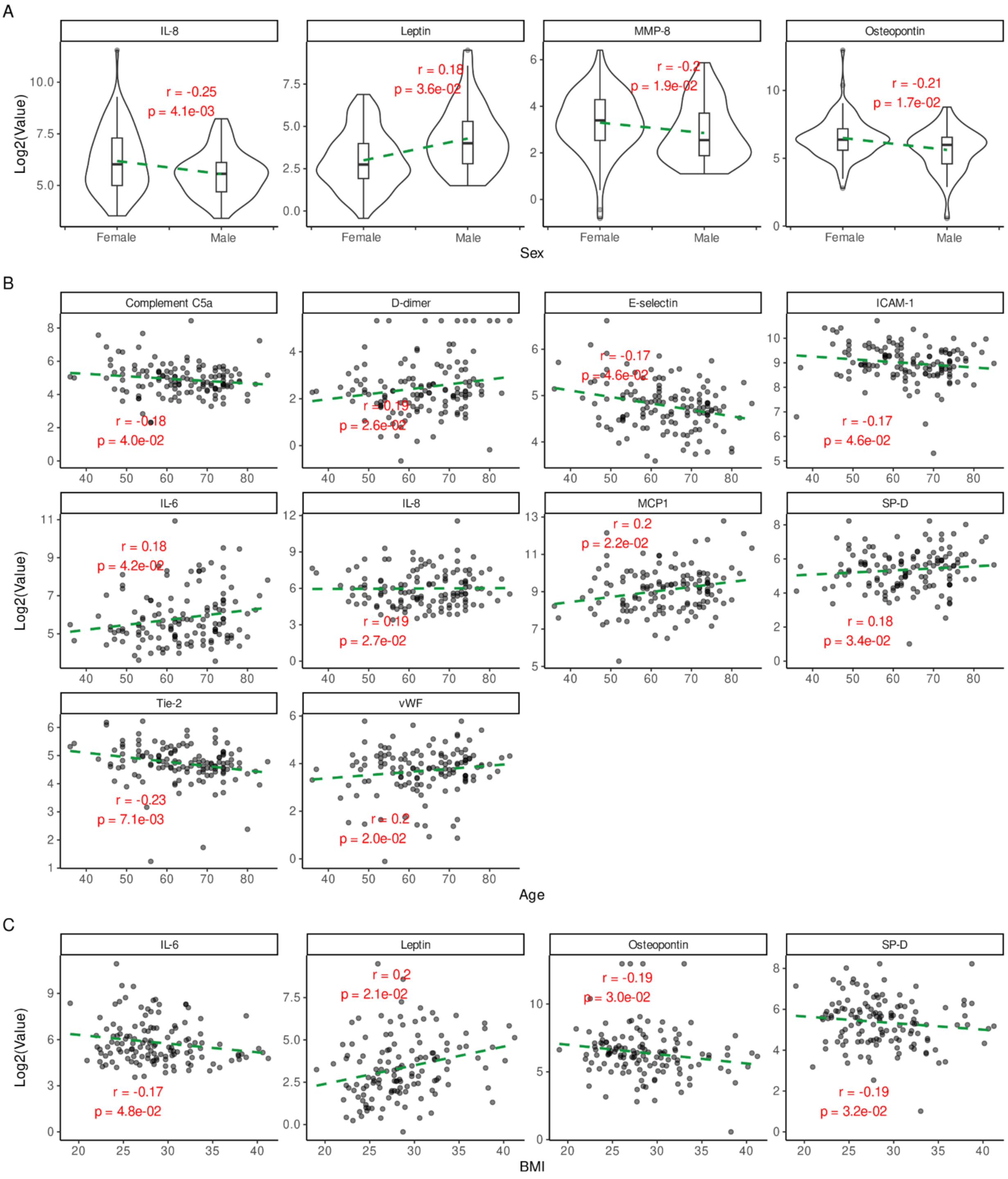

Supplemental Figure 5 : Correlations between Clinical variables and protein levels (Critical). Overview of proteins with significant correlation with clinical parameters. **(A)** Violin-plots visualizing proteins associated with sex in critical COVID-19 patients. The violin plot shows the log2 data distribution of the protein expression data and the boxplot displayed inside the violin indicates the median with interquartile range (IQR). (**B**) Scatter plots visualizing the significant correlation between protein levels and age in critical COVID-19 patients. **(C)** Point scatter plots visualizing the significant correlation between protein levels and BMI in critical COVID-19 patients. . In each plot the x-axis represent the clinical parameter of interest, while the y-axis represents the protein levels after log2 transformation.

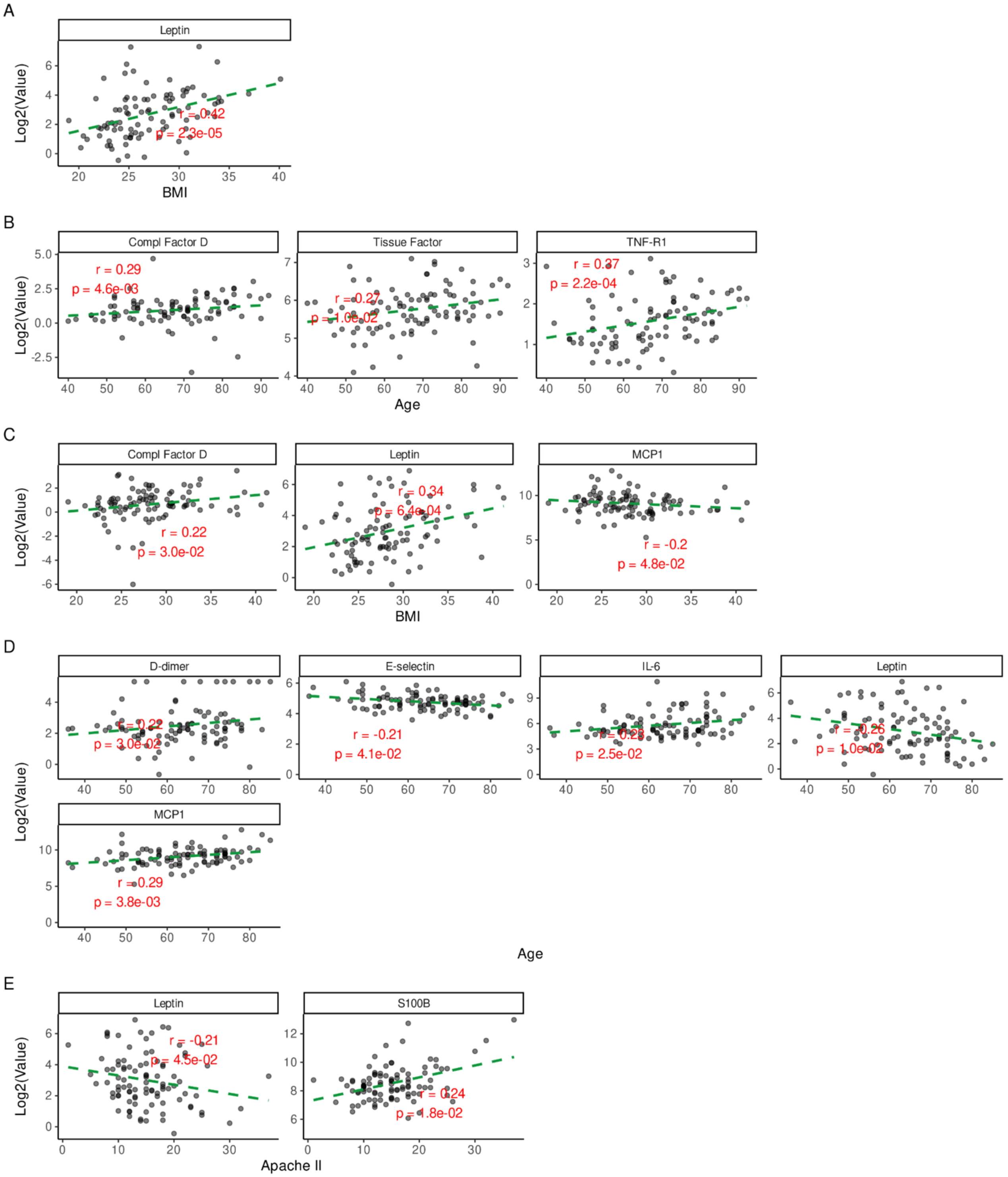

Supplemental Figure 6 : Overview of correlations observed in severe and critical Male

Overview of all significant correlation in both severe and critical COVID-19 male patients. (**A**) (**B**). Scatter plots visualizing the significant correlation observed in severe (Ward) males. **(C) (D) (E)** Scatter plots visualizing significant correlations in critical (ICU) males. The x-axis represents the clinical variable of interest, while the y-axis represents the protein expression after log2 transformation. Figures are annotated with the correlation coefficient (r) and p-value.

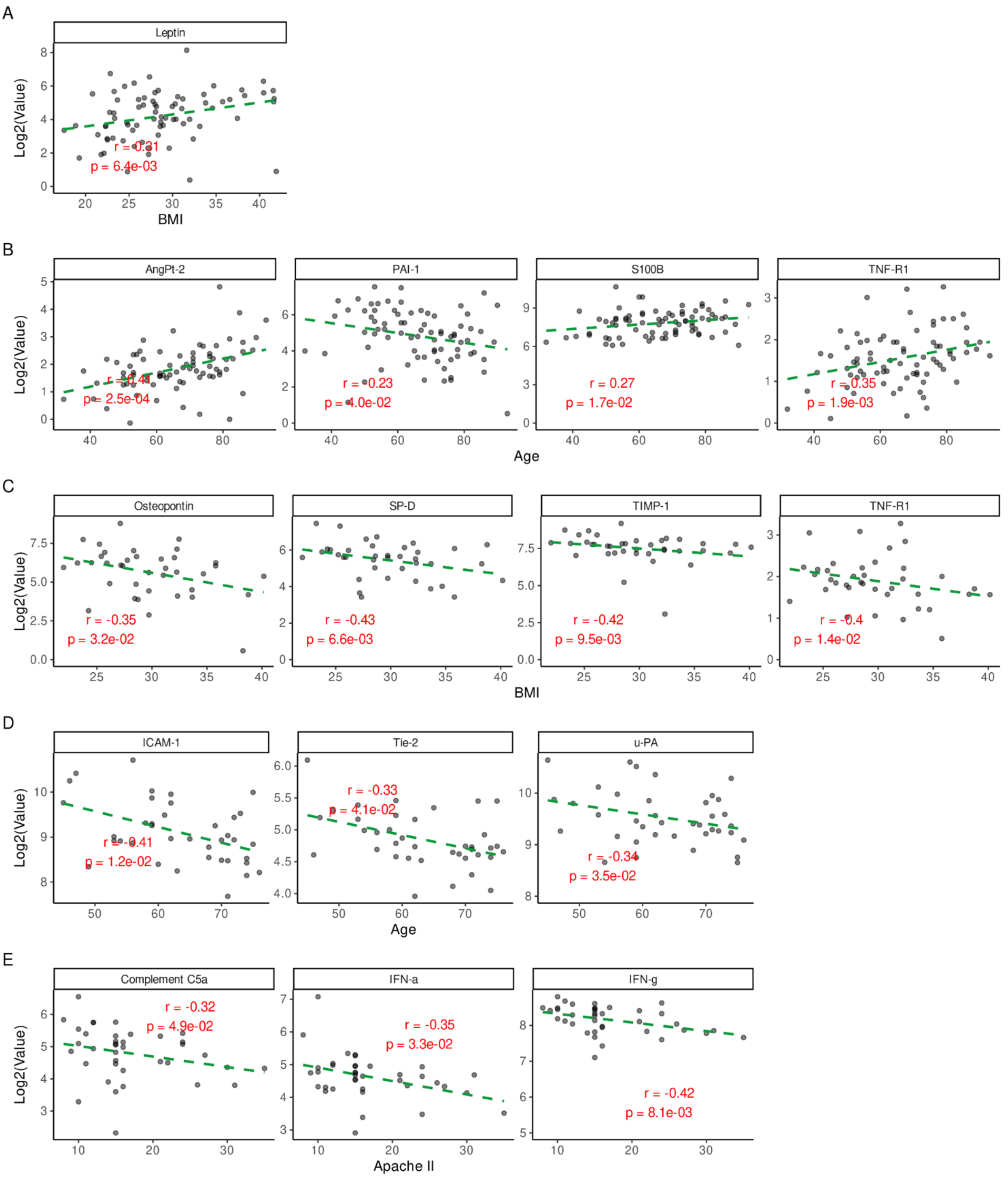

Supplemental Figure 7 : Overview of correlations observed in severe and critical Female

Overview of all significant correlation in both severe and critical COVID-19 female patients. (**A**) (**B**). Scatter plots visualizing the significant correlation observed in severe (Ward) males. **(C) (D) (E)** Scatter plots visualizing significant correlations in critical (ICU) females. The x-axis represents the clinical variable of interest, while the y-axis represents the protein expression after log2 transformation. Figures are annotated with the correlation coefficient (r) and p-value.

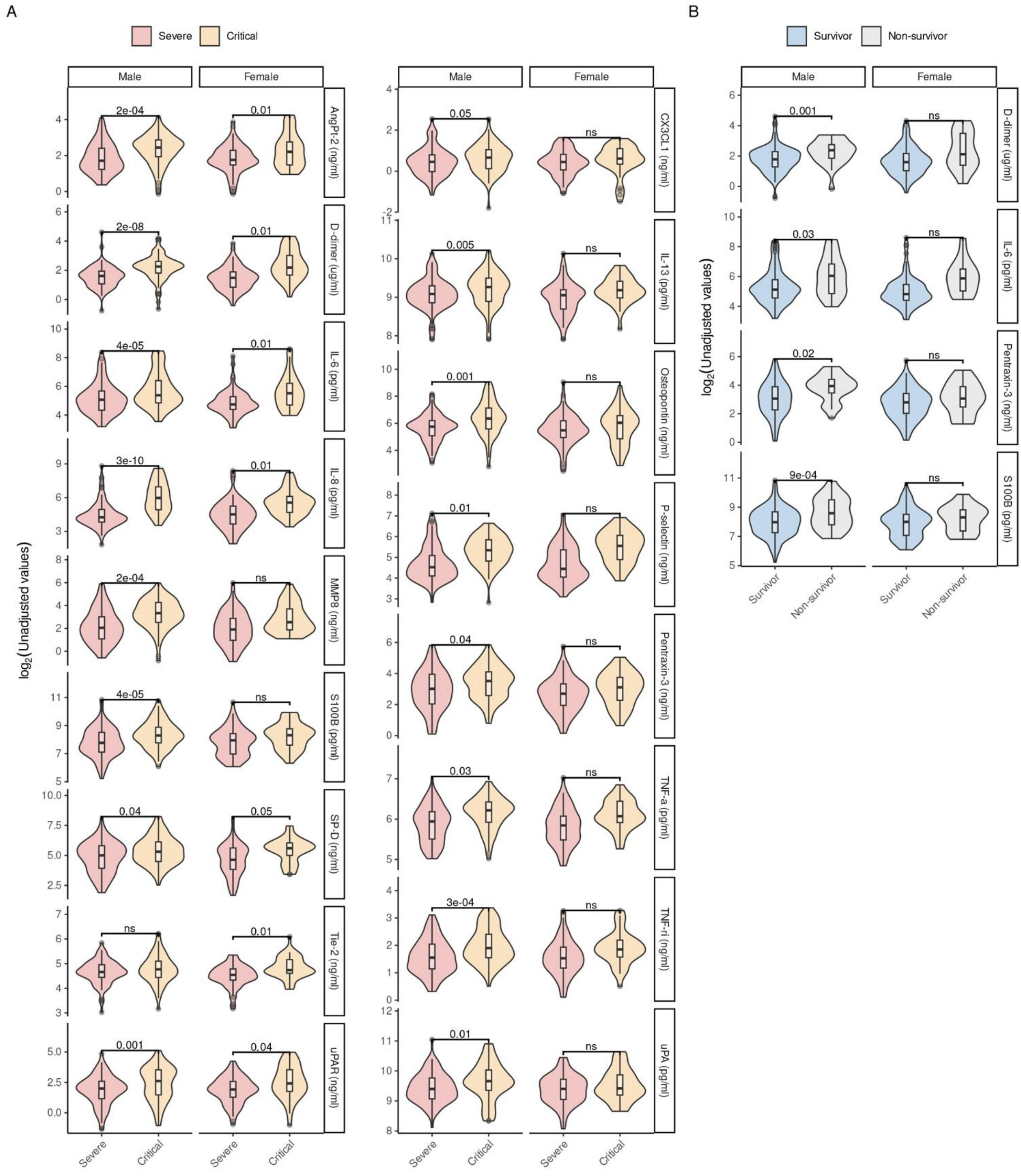

Supplemental Figure 8 : Overview of significant sex-specific proteins. (Unadjusted)
Overview of all proteins identified as sex-specific. Violin-plots visualizing proteins associated with COVID-19 severity (**A**) and mortality (**B**). The violin plot shows the log2 data distribution of the protein expression data and the boxplot displayed inside the violin indicates the median with interquartile range (IQR). The colors represent the different groups based on COVID-19 severity and mortality.

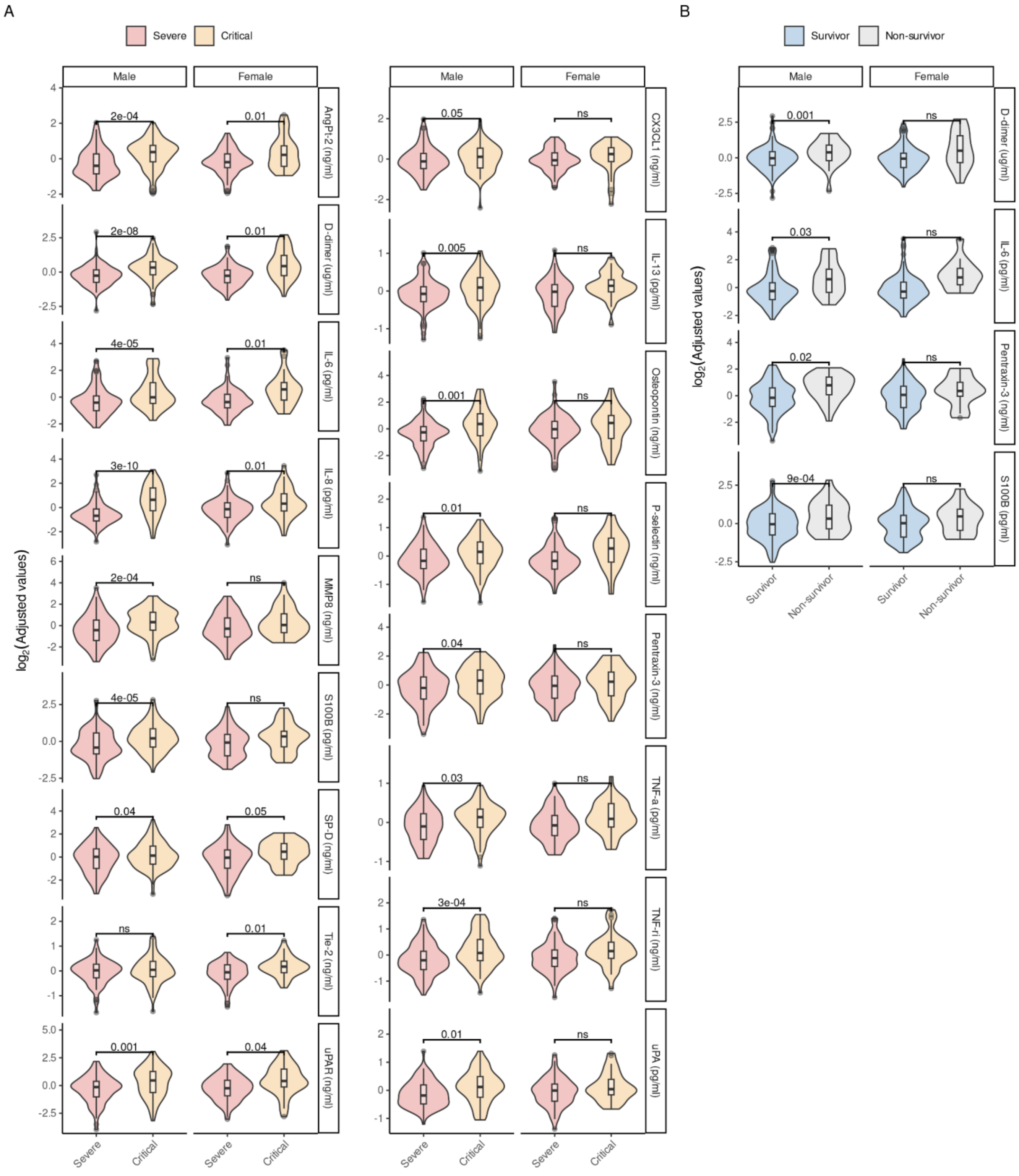

Supplemental Figure 9 : Overview of significant sex-specific proteins. (Adjusted)

Overview of all proteins identified as sex-specific.The violin plot shows the log2 data distribution of the protein expression data based on COVID-19 **(A)** severity and **(B)** mortality. The violin plot shows the log2 data distribution of the protein expression data and the boxplot displayed inside the violin indicates the median with interquartile range (IQR). The colors represent the different groups based on COVID-19 severity and mortality, while the y-axis represents the protein expression after correcting for Sex, BMI, Age, hospital center, diabetes and corticosteroid by using a standard linear model.
